# Presence of viable gram-positive bacteria in blood of patients with inflammatory bowel disease is not affected by treatment

**DOI:** 10.1038/s41598-025-07535-z

**Published:** 2025-06-25

**Authors:** Sanna Davidson, Yunjeong So, Elin Oscarsson, Åsa Håkansson, Klas Sjöberg

**Affiliations:** 1 Department of Clinical Sciences, Malmö, Lund University, Malmö, Sweden; 2 Department of Gastroenterology and Nutrition, Skåne University Hospital, Malmö, 214 28 Sweden; 3 Department of Process and Life Science Engineering, Lund University, Lund, Sweden

**Keywords:** Crohn’s disease, Inflammatory bowel disease, Microbiome, Permeability, Translocation, Ulcerative colitis, Immunology, Microbiology, Gastroenterology

## Abstract

In inflammatory bowel disease (IBD) the pathogenetic process is characterized by dysbiosis, increased permeability, translocation, and immune activation. The aim of the present study was to assess the presence of viable bacteria in the blood of patients with IBD and to correlate the findings with clinical characteristics. The study included 28 patients with Crohn’s disease (CD) (median age 38 years, 50% female, biological treatment in 71%) and 19 patients with ulcerative colitis (UC) (median age 45 years, 33% female, biological treatment in 84%). Identification of viable bacteria in the blood was evaluated by optimized cultivation and Sanger sequencing and for quantification real-time PCR was performed. Viable Gram-positive bacteria were detected in 34 IBD patients (72.3%). There were no associations between the presence of bacteria and gender, antibiotic treatment, intake of alcohol, use of PPI, steroids, or biological treatment. The number of bacterial copies was correlated with higher C-reactive protein (CRP) (*p* = 0.013). In ¾ of the patients, viable bacteria were identified in the blood despite treatment with biologicals, which indicates a vast barrier defect. This observation also indicates that the disease is still active. To obtain a true deep mucosal healing an intact barrier function is required.

## Introduction

Inflammatory bowel disease (IBD) consists of ulcerative colitis (UC) and Crohn’s disease (CD) where UC affects the colon and CD both small intestine and colon. The incidence of IBD is increasing worldwide. In Europe more than 3 million people are affected^1^. IBD is characterized by chronic inflammation of the gastrointestinal tract. Invasive bacteria producing hydrogen sulfide are increased in number while bacteria producing short chain fatty acids are decreased^2–5^. In the intestinal microbiome of IBD patients, there are fewer members from various taxa within the Firmicutes and Bacteroidetes phyla and an increase in the Gammaproteobacteria, meanwhile bacterial species within for example the *Bifidobacterium*, *Lactobacillus*, and *Faecalibacterium* genera are decreased^4^.

The barrier integrity is compromised leading to an increased intestinal permeability. Fyderek et al. found a thinner mucus layer in adolescents with UC and CD in the inflamed site compared to the non-inflamed site and predominance of *Lactobacillus* spp. in the inflamed mucosa of UC and *Streptococcus* spp. in CD^6^. This increased permeability could lead to translocation of bacterial toxins, and possibly also bacterial cells, across the intestinal epithelium with a resulting immune activation^7^.

However, if translocation should occur into the blood of IBD patients this could hypothetically be used as a marker of a disrupted barrier function instead^8^. The presence of bacterial DNA in blood has been reported in patients with CD and UC, both in active disease and remission compared to healthy controls that had no bacterial DNA detected^9^. Bacterial DNA in CD has also been shown as an independent risk factor of flare up after 6 months and a significant predictor of hospitalization, initiation of steroids and switch of treatment^10^. Vrakas et al. found bacterial DNA in the whole IBD-population studied and the concentration was higher in those with active IBD^11^. We have recently revealed the presence of bacterial DNA in the blood of patients with IBD, both in CD and UC^12^.

This leads to the question whether even viable bacteria could penetrate the intestinal wall. The existence of a blood microbiome has been proposed. In 28 healthy individuals the dominant bacterial phyla among non-cultured samples were Proteobacteria 93%, and Firmicutes 2%, while among cultured samples Proteobacteria were found in 48%, Firmicutes in 26%, Actinobacteria in 16%, Bacteroidetes in 3%, and Cyanobacteria in 3%^13^. In accordance with these observations, another study also confirmed that the dominant phyla were Proteobacteria (more than 80%), Actinobacteria (6.7–10%), Firmicutes (3-6.4%) and Bacteroidetes (2.5–3.4%)^14^. Among immune-driven diseases, 20 patients with rheumatoid arthritis (RA) had a different blood microbiome compared to healthy individuals^15^. In 20 patients with psoriasis the group had lower bacterial diversity and richness compared with eight healthy controls^16^. In celiac disease a dysbiotic blood microbiome has been reported^17^. Studies focusing on IBD are scarce^18^. However, the existence of bacterial extracellular vesicles in blood in both IBD and healthy individuals has been observed^19^.

Consequently, the evidence of microbial translocation in IBD is sparse and little is known how this is related to the severity of the disease. Since we have revealed bacterial DNA in blood in patients with IBD the question arises whether also viable bacteria could be present. The aim of the present study was to assess the presence of viable bacteria in the blood and thus the severity in barrier defect of patients with IBD, i.e., CD and UC and to correlate the findings with clinical characteristics.

## Results

### Patient characteristics

The study included 28 patients with CD and 19 patients with UC. Patient characteristics are shown in Table 1. The median age in the whole group was 39 years (IQR 30–54; 46.2% female).

**Table 1:** Patient and clinical characteristics.

	Whole group	Crohns’s disease	Ulcerative colitis
Patients, n (%)	47	28 (59.6%)	19 (40.4%)
Gender (female/male), n	20 / 27	14 / 14	6 / 13
Age, years (median (IQR))	39 (30–54)	38 (30.5–53.8)	45 (29–60)
Age at diagnosis, years (median (IQR))	26.2 (19.3–37.4)	23.2 (19-33.5)	32.3 (22-50.3)
Disease duration, years (median (IQR))	7.8 (2-18.8)	8.7 (3.9–20.3)	4.6 1.2–13)
Montreal A (age at diagnosis), n (%)			
A1 (0–17)	5 (10.6%)	4 (14.3%)	1 (5.3%)
A2 (17–40)	32 (68.1%)	20 (71.4%)	12 (63.2%)
A3 (> 40)	10 (21.3%)	4 (14.3%)	6 (31.6%)
Montreal L (location) n (%)			
L1 (ileal)		10 (35.7%)	
L2 (colonic)		5 (17.9%)	
L3 (ileocolonic)		13 (46.4%)	
Montreal B (behaviour) n (%)			
B1 (nonstricturing, nonpenetrating)		11 (39.3%)	
B2 (stricturing), B3 (penetrating), p (perianal disease)		17 (60.7%)	
Montreal, E (extent) n (%)			
E1 (ulcerative proctitis)			0 (0%)
E2 (left-sided UC)			7 (36.8%)
E3 (extensive UC)			12 (63.2%)
IBD-therapy, n (%)			
None	1 (2.1%)	1 (3.6%)	0 (0%)
5-aminosalicylic acid	14 (29.8%)	1 (3.6%)	13 (68.4%)
Corticosteroids	14 (29.8%)	6 (21.4%)	8 (42.1%)
Chemotherapy	15 (31.9%)	10 (35.7%)	5 (26.3%)
Biological treatment	36 (76.6%)	23 (82.1%)	13 (68.4%)
Use of proton-pump inhibitors, n (%)	12 (25.5%)	9 (32.1%)	3 (15.8%)
Use of antibiotics past two months, n (%)	8 (17.0%)	4 (14.3%)	4 (21.1%)
Use of alcohol four days prior to testing, n (%)	18 (38.3%)	15 (53.6%)	3 (15.8%)
Calprotectin, mg/kg (median (IQR))	379 (181.3–591)	208 (140.5-366.5)	585 (332.8-1326.3)
CRP, mg/L (median (IQR))	2.2 (0.9–5.7)	2.1 (0.9-5.0)	3.1 (0.9–9.7)
Albumin, g/L (median (IQR))	42 (38–44)	41.5 (38–44)	43 (37.5–45)
Hemoglobin, g/L (median (IQR))	135 (124–145)	134 (123.8-145.3)	140 (124-147.5)

Chemotherapy = Azathioprine, 6-mercaptopurine, methotrexate. Biological treatment = adalimumab, infliximab, vedolizumab, ustekinumab, tofacitinib CRP = C-reactive protein.

In the CD group the median age was 38 years (IQR 30.5–53.8; 50% female). Among the CD patients 35.7% presented with ileal disease, 17.9% with colonic disease and 46.4% with ileocolonic disease. Stricturing, penetrating or perianal disease were present in 60.7%. In the UC group the median age was 45 years (IQR 29–60) and 32.6% were female. Extensive UC was present in 63.2% of the patients.

Biological treatment was administered to 76.6% of the participants (71.4% in the CD group and 84.2% in the UC group). Faecal calprotectin was only available in twelve patients with a median value of 379 mg/kg (IQR 181.3–591) in the whole group.

Taking the whole group into account and according to the questionnaire, eleven patients had diarrhoea, five patients had blood in stool, seven patients had diarrhoea with abdominal pain and eight patients had abdominal pain. Two patients reported minor gastrointestinal symptoms and one patient moderate. One patient reported obstipation and one urgency. Six patients did not report any gastrointestinal symptoms, and information was missing for five patients.

## Presence and identification of viable bacteria

Viable bacteria were detected in 34 patients (72.3%) in the whole group, in eighteen patients with CD (64.3%) and sixteen patients with UC (84.2%).

Identified bacteria on genus and species level are shown in Table 2 for genus and Table 3 for species, respectively. Species of the genus *Lactobacillus* was the most found viable bacteria in the whole group of patients (*n* = 10) followed by species of *Bacillus* (*n* = 8), *Staphylococcus* (*n* = 5) and *Micrococcus* (*n* = 4), but the species *Pediococcus pentosaceus*, *Dioszegia hungarica* (yeast), *Micromonospora tulbaghiae* and *Laceyella sacchari*/*Laceyella tengchongensis* were also detected. In 14 patients the cultures were not able to identify.

**Table 2:** Identified genus in the whole group.

Genus	Patients (*N*)
*Lactobacillus* *	10
*Bacillus* *	8
*Staphylococcus* *	5
*Micrococcus* *	4
*Laceylla* *	1
*Micromonospora* *	1
*Pediococcus* *	1
*Dioszegia* (yeast)	1
Not identified	14

* = Gram-positive bacteria.

**Table 3:** Identified species in the whole group.

ID	CD/UC	Sex	Age at dx	Duration	CRP	Bact DNA	CFU/ml	Species
45	CD	F	20.3	9.6	0.69	725	73	*L. plantarum*
								*B. subtilis*
								*S. epidermidis, S. caprae, S. capitis*
49	CD	F	11	45.8	0.9	1423	40	*L. plantarum, L. xiangfangensis*
46	CD	F	16.5	16.8	0.94	1023	51	*L. plantarum, L. pentosus*
33	CD	M	31.8	4.6	2.5	5547	1	*B. amyloliquefaciens*
								*M. luteus, M. yunnanensis*
47	CD	F	67.3	3	3.7	1205	30	*L. plantarum, L. pentosus*
38	CD	F	45.3	8.8	4.4	16900	984	*Dioszegia hungarica*
73	CD	M	37.4	15.6	4.7	990		*S. hominis*
41	CD	F	21.4	12.5	5.7	1433	27	*L. plantarum, L. pentosus*
								*B. amyloliquefaciens*
34	CD	F	26	6.2	16	10,446	1	*M. luteus, M. aloeverae, M. yunnanensis*
								*B. subtilis, B. velezensis, B. amyloliquefaciens*
31	CD	M	24.1	0.3		10,321	16	*S. hominis*
								*Pediococcus pentosaceus*
42	UC	M	17.3	1.8	0.6	846	34	*L. plantarum, L. pentosus*
71	UC	M	60	0.7	0.82	591	1	*M. aloeverae*
56	UC	M	22.5	15.3	0.9	1,017	1	*Laceyella sacchari*
								*B. rhizosphaerae, B. clausii*
55	UC	M	37.3	29.3	2	977	4	*L. plantarum, L. pentosus*
								*B. velezensis, B. amyloliquefaciens*
								*Micromonospora tulbaghiae*
35	UC	F	26.2	21.3	3.6	12,672	2	*S. hominis*
								*M. luteus, M. aloeverae, M. yunnanensis*
57	UC	M	22	23.3	4	1119	1	*B. velezensis*
48	UC	M	27.7	0.6	5.5	1583	16	*L. plantarum, L. pentosus, L. paraplantarum*
72	UC	M	44.2	0.2	9.5	969		*B. velezensis *
40	UC	F	18.2	1.2	67	15,295	22	*L. plantarum, L. pentosus*
								*S. epidermidis*
44	UC	M	32.3	13		1,689	10	*L. plantarum, L. pentosus*

L = Lactobacillus, B = Bacillus, S = Staphylococcus, M = Micrococcus.

Dx = diagnosis, bacterial DNA is stated in copies//µl and CPR levels in mg/L.

In both the whole group and in the CD and UC subgroups, there were no significant association between the presence of viable bacteria and gender, CD or UC, antibiotics past two months, alcohol four days prior to testing, use of PPI, steroids or biological treatment (Table 4). There was a trend towards more smokers in the CD-group with presence of viable bacteria (Table 4). However, no significant association between viable bacteria and smoking was found neither in the group as whole nor in UC.

**Table 4:** Presence of viable bacteria and patient characteristics in the total group.

	CD + UC	*P*-value	CD	*P*-value	UC	*P*-value
Females	14 (41.2)	1.0	9 (50.0)	1.0	5 (31.1)	1.0
Smoking	7 (20.6)	0.17	6 (33.3)	0.062	1 (6.3)	1.0
Antibiotics*	6 (17.6)	1.0	3 (16.7)	1.0	3 (18.8)	0.53
Alcohol**	11 (32.4)	0.20	8 (44.4)	0.25	3 (18.8)	1.0
PPI	7 (20.6)	0.27	4 (22.2)	0.21	3 (18.8)	1.0
Steroids	11 (32.4)	0.73	5 (27.8)	0.38	6 (37.5)	0.55
Biologicals	25 (73.5)	0.70	14 (77.8)	0.63	11 (68.8)	1.0

The numbers are stated and then percentage within parentheses, CD = Crohn’s disease, UC = ulcerative colitis, *antibiotics < 2 months, **alcohol < 4 days, PPI = proton pump inhibitors, biologicals = biological treatment.

### Quantification of bacterial DNA

**Fig. 1:**
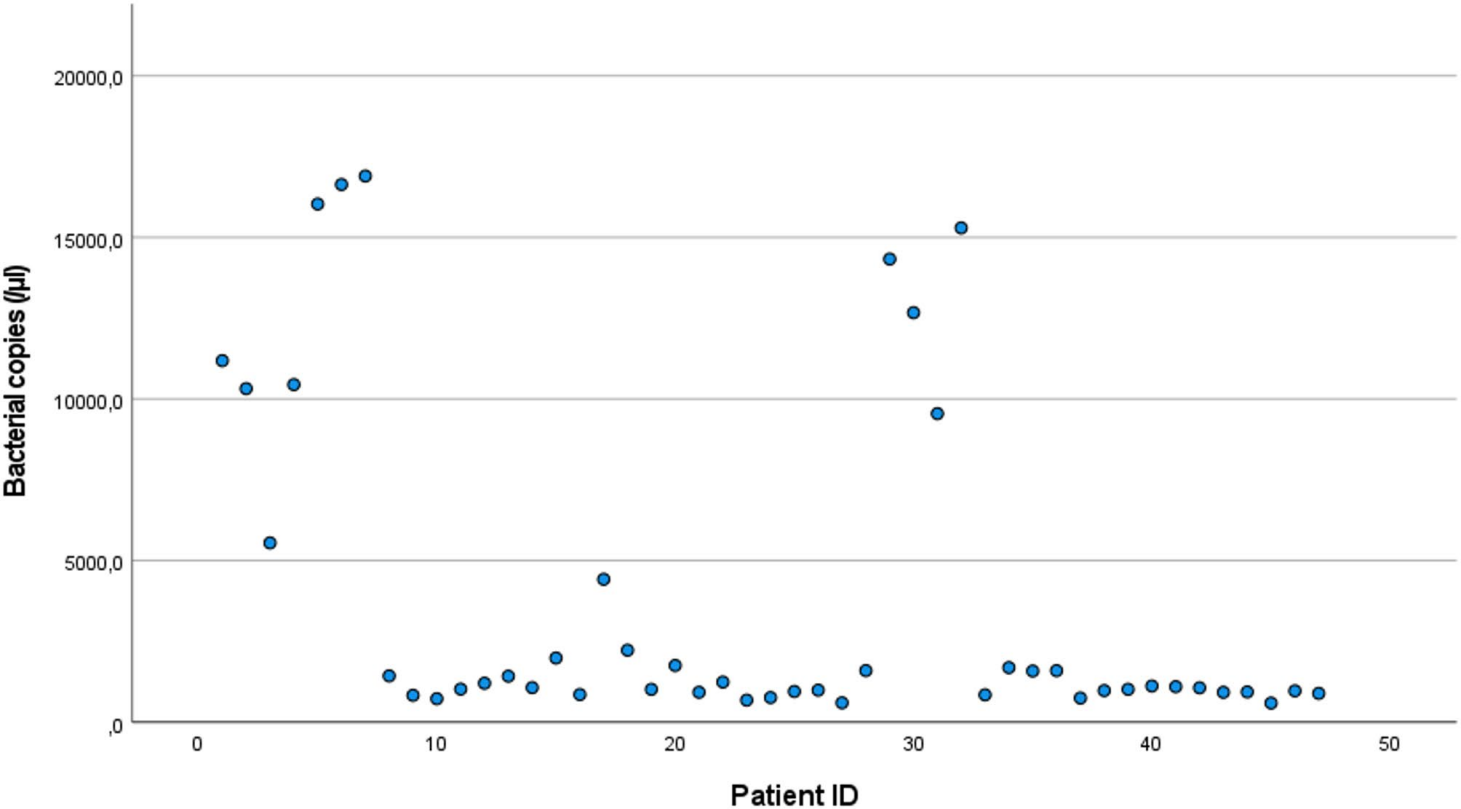
Bacterial copies (/µl) in the whole group of patients with a mean value of 3 887 copies/µl (± 5 194).

**Fig. 2:**
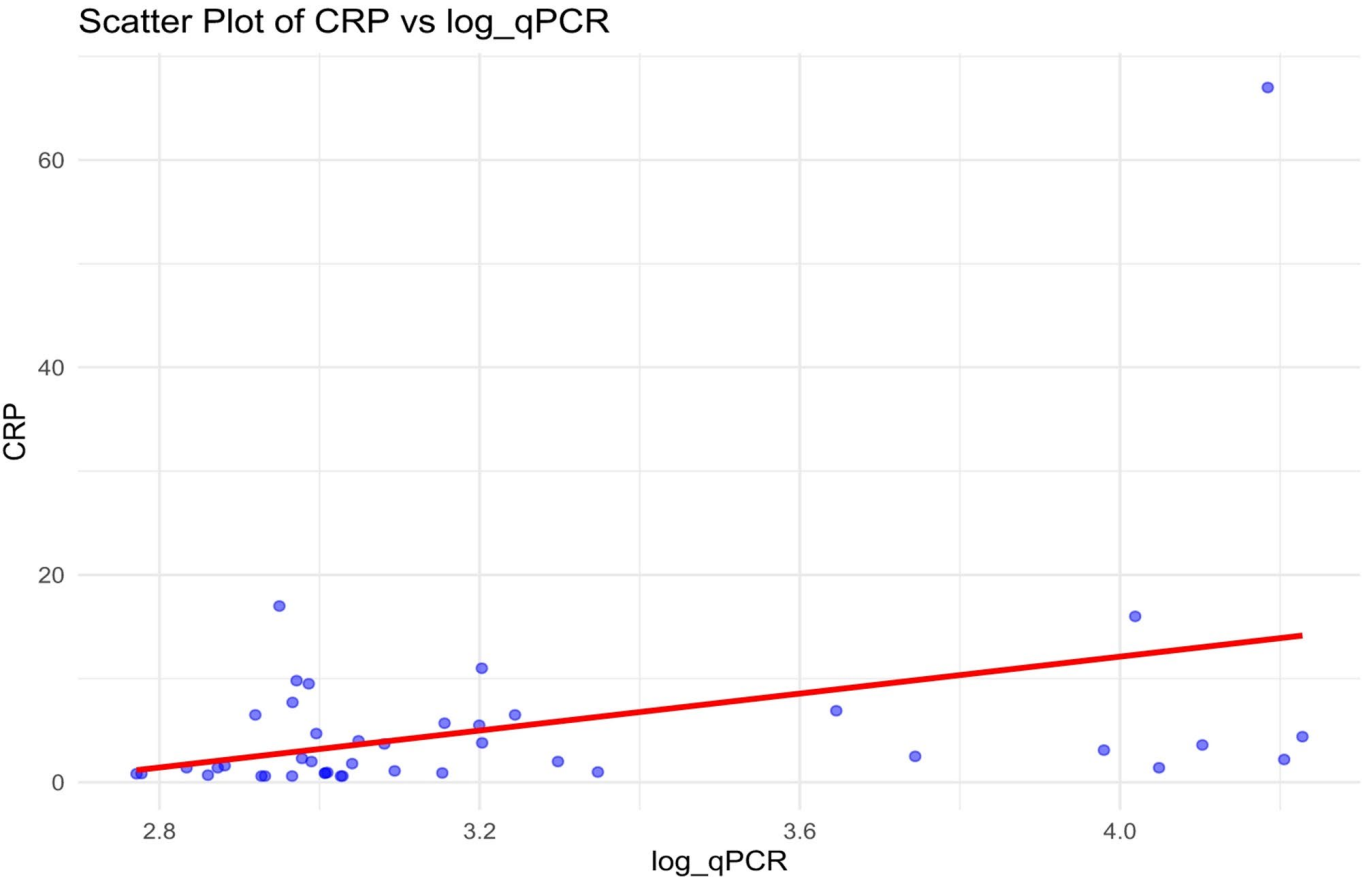
Correlation between CRP and bacterial DNA copies.

As previously reported^12^ bacterial DNA (16SrDNA) was present in all patients (total group; mean 3886.7 copies/µl (± 5194.3), median 1118.6 copies/µl (IQR 925.3-4421.1) (Fig. 1). There was no significant difference between the number of bacterial copies between the CD-group or UC-group (*p* = 0.49). In the total group, the number of bacterial copies was significantly correlated with higher CRP (correlation coefficient (CC) 0.38, *p* = 0.013) (Table 5 and Fig. 2) and there was a trend towards higher CRP in both the CD group (CC = 0.360, *p* = 0.071) and the UC group (CC = 0.447, *p* = 0.072). In the total group there was also a trend for correlation between lower albumin and number of bacterial copies (CC=-0.285, *p* = 0.064). There was no significant difference in the number of bacterial copies between the five different groups of IBD-therapies (data not shown; H = 3.061, *p* = 0.55).

Furthermore, no association could be found with age, BMI, age at diagnosis, duration, pack-years of smoking, calprotectin, hemoglobin while CRP (significant) and albumin (inverse trend) were associated (Table 5). Furthermore, no differences were found in relation to behaviour in CD (*p* = 0.47), and not regarding location either, not for IBD in general (H = 3.061, *P* = 0.55) and neither in CD (H = 1.324, *p* = 0.52) nor in UC (*p* = 0.67).

**Table 5:** Correlation between bacterial copies and patient characteristics and biomarkers in the whole group, CD group and UC group.

	CD + UC		CD		UC	
	CC	P-value	CC	P-value	CC	P-value
Age	0.002	0.992	0.130	0.510	-0.173	0.478
BMI	0.195	0.189	0.134	0.498	0.338	0.157
Age at diagnosis	-0.148	0.320	-0.130	0.510	-0.158	0.519
Disease duration	0.197	0.184	0.283	0.145	-0.035	0.887
Pack-years of smoking	-0.212*	0.152	-0.273	0.160	-0.381*	0.108
Calprotectin	-0.350*	0.265	-0.587	0.221	-0.184	0.727
Hemoglobin	-0.244	0.115	-0.346	0.083	-0.077	0.768
CRP	0.377*	0.013	0.360*	0.071	0.447*	0.072
Albumin	-0.285	0.064	-0.325	0.105	-0.212	0.414

*Spearman’s correlation, CC = correlation coefficient.

**Fig. 3:**
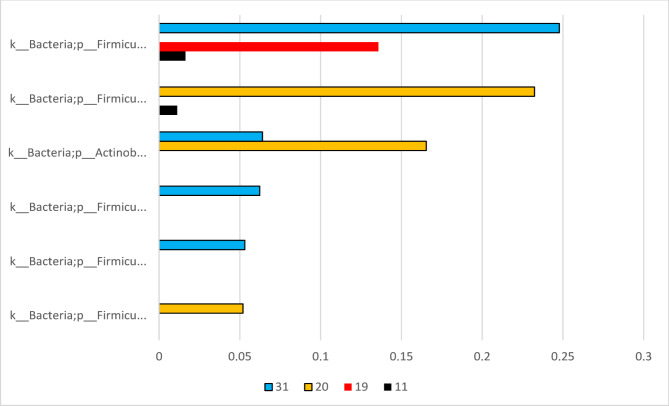
Relative abundance of bacterial DNA in the control group. Relative abundance levels below 0.1% have been omitted.

Out of the 33 analyzed samples in the control group, five yielded a sequencing depth of over 10,000 reads, indicating a high level of coverage suitable for further analysis. The No Template Control (NTC), which is expected to contain no sequences, had 980 reads – suggesting minor contamination or background noise. All values represent the number of sequencing reads per sample. The bacteria DNA in the control group predominantly originated from the Firmicutes phylum. The other two identified phyla were the Actinobacteria phylum and to a very small extent the Proteobacteria phylum, the last less than 0.1%. This differs significantly from the presence of bacterial DNA in the IBD group (*p* = 0.0058). See Fig. 3.

## Discussion

This study detected viable bacteria in blood of patients with moderately active IBD coming for a regular check-up visit. Only phyla belonging to Gram-positive bacteria (and in one patient yeast) were identified. The most dominant genus was *Lactobacillus* followed by *Bacillus*, *Staphylococcus* and *Micrococcus*. Besides the previous observation of bacterial DNA in blood^12^ also viable bacteria could thus be found in the blood in 72.3% of the IBD patients. This means that the barrier integrity in the intestine is malfunctioning to a higher degree than previously anticipated.

Bacterial DNA could be found in all patients. This differs significantly from the occurrence in healthy individuals (15%). Even though the patients only had minor symptoms they still had translocation indicating that the disease was more active than anticipated by the clinical findings. Maybe translocation could be used as an extra biomarker indicating active disease. Compared to the number of live bacteria in sepsis estimated by the numbers of colony forming unit (CFU), the numbers in IBD in the present study seem to be comparable^20^. However, based on bacterial DNA in healthy individuals, the magnitude differs. In a study on healthy individuals from seven European countries the numbers differed between 80 and 160 copies/ml (mean value 122 copies/ml)^21^. In another study the number of copies in healthy blood donors was 4.0 × 10^7^ copies/ml^14^. This difference is too large to be explained solely by variations between countries and may instead arise from differences in experimental controls and decontamination strategies. The outcome in the present study of 3 887 copies/µl is much higher than the first study, but in line with the second. Previous studies have shown that presence of bacteria in blood is a rare phenomenon in healthy individuals, occurring at a low frequency^22^. In healthy individuals, microbial DNA originates from circulating commensals that coexist asymptomatically with the host and exhibit immunomodulatory phenotypes. These properties are critical in determining whether bacteremia leads to asymptomatic coexistence or progresses to sepsis^22,23^.

However, two studies have reported presence of viable bacteria in hospitalized patients with IBD. One study has retrospectively collected data on blood stream infections in IBD patients during their stay in hospital. Both Gram-positive and Gram-negative bacteria were identified. The most prevalent bacteria were *Staphylococcus epidermidis* among the Gram-positive and *Escherichia coli* and *Enterobacter* among the Gram-negative. The most common reason for septicemia was catheter borne infections^24^. The next study scrutinized the bacterial origin in infections in patients with IBD but also other infections. Also, in this study the most prevalent bacterium was *Staphylococcus epidermidis*^25^. The crucial difference between these two studies and the present is the fact that these two studies report which bacteria that were found in patients that developed septicemia while the present study reports findings of viable bacteria in otherwise fairly asymptomatic patients attending the out-patient clinic for a routine check-up.

In the present study, the most found viable genus was *Lactobacillus* – i.e., in 10 out of 34 patients consisting of *Lactiplantibacillus plantarum*,* Lactiplantibacillus pentosus.*, and *Lactiplantibacillus paraplantarum*. This is a group of closely related Gram-positive bacteria all belonging to the *L. plantarum* group, considered to have beneficial effects. However, a possibility that species of this genus could result in bacteremia has been contemplated. Reports of *Lactobacillus* bacteremia, often linked to severe underlying conditions, suggest it may serve as a marker of serious disease rather than being fatal itself. A 15-year case series^26^ involving 45 immunocompromised patients reported only one death due to *Lactobacillus* bacteremia. In southern Finland, eight cases of *Lactobacillus* bacteremia were found between 1989 and 1992, where five patients had severe comorbidities^27^. In other words, bacteriemia from *Lactobacillus* seems to be a rare phenomenon predominantly affecting immunocompromised patients. *Lactobacillus* strains also have an immunoregulatory effect and can stimulate several cytokines with different roles (TNF-alfa, IFN-gamma, IL-1beta, IL-10) in human peripheral blood mononuclear cells from healthy donors, where *Lactiplantibacillus plantarum* was more potent than *Lactobacillus acidophilus*^28^. It can be speculated whether *Lactobacillus* could play a regulatory role in the disease pathogenesis and act as a biomarker or if it is just an innocent bystander just passing through.

The second most dominant genus in the study was *Bacillus*, an endospore-forming Gram-positive bacteria, widely spread in nature but also in fermented foods like kimchi^29^. The *Bacillus* species *Bacillus velezensis*, *Bacillus amyloliquefaciens* and *Bacillus siamensis* found in the present study form an operational group (Operational Group *Bacillus amyloliquefaciens* (OGB*a*) and are often used as plant growth-promoting bacteria. *Bacillus velezensis* has been shown to have probiotic potential and may reduce DSS-colitis, inhibit growth of *Clostridium difficile* and *Salmonella* but instead promote growth of *Verrucomicrobia*^30,31^. The current knowledge does not support the idea of *Bacillus* being a potential trigger of IBD. Instead, its beneficial effects and harmless appearance could explain the fact that no danger signals are secreted because of its presence in the bloodstream.

*Micrococcus* was found in four patients and is part of the commensal flora, especially in the skin. The species *Micrococcus aloeverae*, *Micrococus luteus* and *Micrococcus yunnanensis* form the *M. luteus* group but these species have also been proposed to be reclassified as heterotypic synonyms of *Micrococcus luteus*^32^. The incidence of *Micrococcus luteus* bacteremia was as low as 13 per 100,000 in a Chinese study^33^ and mostly found in immunocompromised patients. *Bacillus* and *Micrococcus* are both among the organisms most described as contaminants^34^. It is not very likely that they could have any impact on disease onset or course.

Only coagulase-negative staphylococci (CNS) were found in the present study. *S. epidermis*,* S. hominis and S. capitis* form the *S. epidermis* group^35^. CNS are often regarded as contaminants from the skin and one study showed that only 12.4% of the CNS blood cultures were clinically significant^36^. Our findings of staphylococcus in the blood could very well be contaminants from the skin.

In contrast, no viable Gram-negative bacteria were found in the bloodstream. This is a peculiar observation. However, in IBD several antibodies against Gram-negative bacteria have been identified, such as for example against the outer-membrane porin C of *Escherichia coli* in CD^37^, against a *Pseudomonas* fluorescens-associated sequence I2 in CD but to a minor extent also in UC^38^. These bacteria could have been eliminated by the immune system quicker while the commensal bacteria are allowed to persist viable in the circulation for a longer time. Maybe viable Gram-negative bacteria could have been identified in the blood stream if the patients had had a more active disease.

In our study, no associations between viable bacteria and gender, CD or UC, antibiotics past two months, alcohol four days prior to testing, use of PPI, smoking, steroids, or biological treatment were found. The levels of CRP, albumin and hemoglobin were fairly normal indicating that the inflammatory activity was not too high. Only 12 patients managed to leave fecal samples for calprotectin but the levels were consistent with a moderate inflammatory activity. Bacteria were present in both CD and UC irrespective of biological treatment or not. In other words, this treatment that is effective in reducing the symptom burden does not affect the barrier dysfunction, something that could explain the need for continuous treatment. Translocation seems to be a general finding that all IBD patients could have in common.

The present study has several limitations. Notably, we did not assess viable bacteria in healthy controls, only bacterial DNA using Illumina sequencing. However, this method detects DNA from both live and dead bacteria, and still revealed a markedly lower bacterial load in the blood of healthy individuals compared to IBD patients. This suggests that the observed differences are not solely due to viability, but reflect an overall lower presence of bacterial DNA - regardless of viability - in healthy individuals. Even though less likely, presence of viable bacteria could still be found in healthy individuals as well^14^. Regarding *Lactobacillus*, contamination from the oral mucosa through transient bacteremia from the oral cavity after toothbrushing seems less likely since this often occurs within 5 min after oral procedures and disappears after 20 min^39^. The study did not record the intake of probiotics or food products containing *Lactobacillus* spp. However, *Lactobacillus* bacteremia is uncommon and is rarely caused by probiotic intake^40^. Although several of the detected species are regarded as commensals, correlations with oral or gut and mucosal flora were not carried out in the present study, which could have been helpful in assessing the blood flora. The evaluation of disease activity was restricted by the small number of calprotectin samples (available in only 12 out of 47 patients) and the lack of biopsies. The patient group consisted mostly of patients with advanced IBD, something that must be taken into consideration. This could hypothetically lead to a falsely positive outcome. However, ¾ of the patients were treated with biologicals and they still had bacterial contamination indicating that even with reduced disease activity the bacteria were still present. A strength is the consecutive sampling of IBD patients coming for a regular check-up. Thus, this cohort represents a real-world scenario making it more likely that the findings are generalizable.

## Conclusions

Since even viable bacteria can penetrate the intestinal wall the barrier function seems to be vastly affected. While the DNA chain can be measured in nm the bacterial cell can be measured in µm, i.e., 1 000 times bigger. In IBD a reduced mucus layer and an increased permeability over the epithelium have been reported^6^. The presence of both bacterial DNA and viable bacteria will certainly contribute to the immune activation why further studies focusing on this phenomenon are warranted.

## Patients and methods

### Patients and study design

The study included 47 consecutive patients with either Crohn’s disease (CD, *n* = 28) or ulcerative colitis (UC, *n* = 19) controlled at the outpatient clinic coming for regular check-ups at the Department of Gastroenterology and Nutrition at Skåne University Hospital, Malmö, Sweden during spring 2019. Out of the 47 included patients, 33 of these were included in our previous study^12^. One of the included patients was admitted to the hospital due to a flare-up. Patients on ongoing antibiotics during testing and patients with only ulcerative proctitis were excluded from the study.

Patient data were retrieved using a standardized questionnaire including age, gender, body mass index (BMI), smoking, disease duration, disease behavior and location (according to the Montreal classification), symptoms, IBD-therapy, use of proton-pump inhibitors (PPIs) and antibiotics and alcohol intake the four past days. Date of diagnosis was defined as the date of confirmed histological diagnosis. For determination of inflammatory activity in IBD, the levels for CRP, calprotectin, albumin and hemoglobin were used.

For comparison of presence of bacterial DNA, a group of healthy volunteers was recruited consisting of 33 persons, mean age 24.6 years (26 women, 7 men) range 22–39 years.

### Blood sampling and cultivation

Standard procedures with regard to antiseptic sampling and sterile equipment were applied. Three peripheral blood samples per patient were collected in 3,5 ml serum SST tubes without any additives. The tubes were delivered to the Department of Process and Life Science Engineering, Faculty of Engineering, Lund University, for immediate analysis. Before cultivation, the three blood tubes from each patient were transferred to a sterile 45 ml tube and repeatedly pipetted with a serological pipette using a sterile 10 ml tip to break clots into small pieces. The samples were then sonicated in a Millipore ultrasonic bath for 1 min, to obtain thoroughly homogenized samples. Viable counts were performed on blood from each patient. 100 µl were spread with sterile glass beads on duplicate plates for lactic acid bacteria (De Man, Rogosa and Sharpe agar (MRS), Merck Millipore, Darmstadt, Germany, incubated at 37 °C for 3–15 days), total count (Tryptic soy agar (TSA), Fluka, Missouri, USA, incubated at 37 °C for 3–15 days), for cultivation of fastidious organisms, especially with regard to pathogens and hemolytic bacteria (Blood Agar (BA), Thermo Fisher Scientific, Germany, incubated at 37 °C for 3–15 days), *Enterobacteriaceae* (Violet Red Bile Dextrose agar (VRBD), Merck Millipore, Darmstadt, Germany, incubated at 37 °C for 1–3 days) and yeast and mold (Malt soy agar (MA), Sigma Aldrich, India, incubated at room temperature for 7–21 days). All samples were incubated both aerobically and anaerobically using Anaerocult^®^ A (Merck Millipore, Darmstadt, Germany). All plates were counted after the expected incubation time (first specified time). If no colonies were observed, the plates remained incubated for detection of potential slow-growing microorganisms in human blood. The plates were re-evaluated every third day for TSA, BA, MRS and VRBD, and every week for MA throughout the prolonged incubation time (second specified time). At growth, randomly picked colonies were isolated, re-streaked for purity and stored in freezing media at – 80 °C for further analysis.

### Sanger sequencing

DNA extraction from the isolates was performed using the glass bead beating method^41^. In short, approximately 10 µl of the cultures was picked with a loop and added into a 1.5 ml sterile tube with 1 ml MilliQ water and 10 glass beads. The tubes were shaken using an Eppendorf Mixer 5432 (Eppendorf, Hamburg, Germany) for 30 min at 4 °C. The DNA was separated by centrifugation at 14,000 g for 1 min and the supernatant was used as template in the subsequent polymerase chain reaction (PCR).

The 16S rRNA genes were amplified using the forward primer ENV1 (5’-AGA GTT TGA TII TGG CTC AG-3’) and the reverse primer ENV2 (5’-CGG ITA CCT TGT TAC GAC TT-3’) (Eurofins Genomics, Ebersberg, Germany) with TopTaq DNA Polymerase (Qiagen, Netherlands) in an Eppendorf 5333 Mastercycler Thermal Cycler (Eppendorf, Hamburg, Germany) according to the manufacturers’ instructions. PCR products were confirmed by gel electrophoresis and if unsuccessful using ENV1 and ENV2, the samples were amplified with ITS1F (5’- CTT GGT CAT TTA GAG GAA GTAA-3’) and ITS4 (5’-TCC TCC GCT TAT TGA TAT GC-3’) targeting the 18 S rRNA gene for identification of fungi. To confirm the reaction, the amplification products (1.5 µl) were run on a 1.5% (w/v) agarose gel in TAE buffer (89 mM Tris, 89 mM boric acid, 2.5 mM EDTA, pH 8.3). The gel was run at 120 V for 60 min and stained with GelRed (Biotium, USA) according to manufacturer’s instructions. The PCR products were subsequently sent for sequencing at Eurofins Genomics (Ebersberg, Germany). The sequences were trimmed to between 590 and 788 bp depending on sequence quality and compared to type strains at the Ribosomal Database project (RDP) by Seqmatch^42^.

### Analysis of control group

Total DNA from whole blood samples was extracted using Illumina Flex Lysis Kit (Illumina, CA, USA) according to the manufacturer’s protocol. Then, 16 S metagenomics library preparation was performed using Quick-16 S Plus NGS Library Prep Kit (V3-V4, UDI) (Zymo Research Corp., Irvine, CA, USA). The final library of 6 pM with 15% PhiX spike-in was loaded on a MiSeq sequencer using Miseq reagent kit V3 (600 cycle) (Illumina, CA, USA).

Sequence data analysis was performed using an open-source bioinformatics tool Qiime2^43^. Briefly, adapter and primer sequences were trimmed off using cutadapt plugin^44^. The sequences were further processed with dada2 plugin^45^. The identified amplicon sequence variants (ASVs) were used for further analysis. For the classification of the bacteria, the Naïve Bayes classifier was trained on the V3-V4 region of reference sequences from Greengenes 13_8 (99% sequence similarity) using the QIIME2 plugin feature-classifier^46^.

### Real-time PCR (RT-PCR)

Frozen blood samples were thawed at room temperature, transferred to sterile 50 ml tubes and repeatedly pipetted with a serological pipette using a sterile 10 ml tip, in order to break clots into small pieces. 1 ml of the blood sample was then added to a sterile 1.5 ml tube with glass beads. 100 µl of 1% EDTA and 50 µl proteinase K (600 mAU/ml) (Qiagen, Germany) were added to each tube to increase the yield of bacterial DNA. All samples were put in a mixer (Eppendorf 5432, Hamburg, Germany) at 4 °C for 30 min and centrifuged at 14,000 g for 2 min. 200 µl of the supernatant was collected for DNA extraction using an EZ1 Advanced XL inserted with a bacterial card (Qiagen, Germany) and the EZ1 DNA tissue kit (Qiagen bioinformatics, Aarhus, Denmark) according to the manufacture’s instruction. The extracted DNA was used as a template in the subsequent polymerase chain reaction.

DNA was amplified in a Mastercycler (Eppendorf) using the following program: 94 °C for 3 min, followed by 35 cycles consisting of 94 °C for 1 min, 50 °C for 45 s, 72 °C for 2 min, and a final extension of 72 °C for 10 min. Gel electrophoresis was run on PCR products as mentioned above to confirm the presence of amplified DNA. Samples showing positive bands on gels were further analyzed with real-time PCR.

The number of 16S rDNA copies in whole blood samples were measured by RT-PCR following the procedures previously described by Karlsson et al.^47^. In short, each reaction contained 10 µL 2xRotor-Gene SYBR Green PCR Master Mix (Qiagen) together with 0.5 µmol/L of the primers Y1 (5’-TGG CTC AGG ACG AAC GCT GGC GGC-3’) and Y2 (5’-CCT ACT GCT GCC TCC CGT AGG AGT-3’)^48^, 2 µL DNA template and RNAse-free water, resulting in a final volume of 20 µL. All samples, negative controls and standards were run in triplicates using a Rotor-Gene Q (Qiagen) with a program of 95 °C for 10 min, followed by 40 cycles of 95 °C for 10 s (denaturation), 60 °C for 15 s, and 72 °C for 20 s (annealing and elongation). Absolute amount of 16 S rRNA genes was determined based on calculations using standard curves with known DNA concentrations and Rotor-Gene Q Series Software 1.7 (Qiagen), R2 > 0.998. The standard curve was created by using a serial 10-fold dilution of cloned PCR products from *Streptococcus pseudopneumoniae* CCUG 49455T corresponding to 100 to 109 cells. The number of bacteria was expressed as copies/µl blood.

C-reactive protein (CRP), albumin, hemoglobin and calprotectin were analyzed according to established procedures at the Department of Clinical Chemistry at Skåne University Hospital, Malmö.

### Statistics

Statistical testing was performed in SPSS (version 28). Fischer’s exact test and chi-2 was used for categorial variables. Pearson’s correlation was used for normally distributed data and Spearman’s correlation for non-normally distributed data. Mann Whitney u-test was used for comparison of quantitative non-parametric data and Kruskal Wallis-test was used for association between continuous and ordinal data. p-values ≤ 0.05 were considered significant.

## Data Availability

The data that support the findings of this study are available on request from the corresponding author. The data are not publicly available due to privacy or ethical restrictions.
